# RNA Interference Mediated Interleukin-1*β* Silencing in Inflamed Chondrocytes Decreases Target and Downstream Catabolic Responses

**DOI:** 10.1155/2016/3484961

**Published:** 2016-03-17

**Authors:** Kyla F. Ortved, Bethany S. Austin, Michael S. Scimeca, Alan J. Nixon

**Affiliations:** Department of Clinical Sciences, Cornell University, Ithaca, NY 14853, USA

## Abstract

Posttraumatic activation of the catabolic cascade plays a major role in degradation of cartilage. Interleukin-1*β* (IL-1*β*), a primary instigator in the catabolic axis, is upregulated in chondrocytes following injury. IL-1*β* activates key degradative enzymes, including MMPs and aggrecanases, and other proinflammatory mediators such as PGE_2_ which contribute to ECM breakdown. Posttranscriptional silencing of IL-1*β* by RNA interference (RNAi) may drive a reduction in IL-1*β*. We hypothesized that transduction of chondrocytes using rAAV2 expressing a short hairpin RNAi motif targeting IL-1*β* (shIL-1*β*) would significantly decrease IL-1*β* expression and, in turn, decrease expression of other catabolic enzymes. Chondrocyte cultures were transduced with rAAV2-tdT-shIL-1*β* in serum-free media. The fluorescent protein, tdTomato, was used to determine transduction efficiency via flow cytometry and fluorescent microscopy. Cells were stimulated with lipopolysaccharide (LPS) 48 hours following transduction. After 24-hour stimulation, supernatants were collected for cytokine analysis, and cells lysed for gene expression analysis. IL-1*β* knockdown led to significantly decreased expression of* IL-1β*,* TNF-α*, and* ADAMTS5*. PGE_2_ synthesis was also significantly downregulated. Overall, effective silencing of IL-1*β* using rAAV2 vector expressing a short hairpin IL-1*β* knockdown sequence was shown. Additionally, significant downstream effects were evident, including decreased expression of* TNF-α* and* ADAMTS5*. Targeted silencing of catabolic cytokines may provide a promising treatment avenue for osteoarthritic (OA) joints.

## 1. Introduction

Osteoarthritis (OA) is a prevalent, debilitating disease that currently lacks any effective treatment and often culminates in total joint replacement surgery. It is characterized by progressive degradation of the extracellular matrix (ECM) leading to joint swelling, reduced mobility, and pain. Chondrocytes are the sole cell type responsible for maintaining the integrity of the ECM, and disruption of chondrocyte metabolism following trauma to the articular surface is considered pivotal in deterioration of the osteoarthritic joint. Perturbations in gene expression patterns of chondrocytes, including increased expression of catabolic cytokines and degradative enzymes, have been shown to cause slow destruction of the ECM [1]. Interleukin-1*β* (IL-1*β*), a proinflammatory cytokine, is considered a primary instigator in cartilage degradation [2–4] and is upregulated in OA chondrocytes and synovial fluid from OA joints [5]. IL-1*β* acts through autocrine and paracrine signaling pathways to increase synthesis of degradative enzymes, including MMPs and aggrecanases, which actively break down the ECM [6]. Simultaneously, IL-1*β* decreases expression of collagen type II and inhibits aggrecan synthesis [7, 8]. Inflammatory mediators, such as prostaglandin E_2_ (PGE_2_) [9] and nitric oxide (NO) [10], are also increased by IL-1*β* and likely play a significant role in the pain response associated with OA.

The beneficial role of IL-1 receptor antagonist protein (IL-1Ra) has received significant attention as it decreases the degradative effects of IL-1*β* through competitive binding of IL-1 receptors [11–13]. A potential impediment to IL-1Ra therapy is the requirement of 100-fold, or greater, excess of IL-1Ra over IL-1 to achieve effective antagonism [14]. Similarly, maintaining sufficient IL-1Ra concentrations in the synovial fluid is difficult, requiring daily injections and pushing against the positive feedback loop exhibited by IL-1*β* [15, 16]. Posttranscriptional silencing of IL-1*β* through RNA interference (RNAi) may offer a superior method of controlling the catabolic cascade involved in joint injury and OA. Small interfering RNAs (siRNAs) were originally investigated as therapeutic modalities. However, DNA-based sustainable expression of short hairpin RNAs is clearly required for progression to clinical therapy, and viral based gene delivery provides the most robust method for target cell cytokine suppression.

A gene therapy approach for treating OA is attractive as the disease often affects a single joint that can be treated locally with an intra-articular injection of a vector overexpressing a therapeutic transgene. Sustained, vector-mediated knockdown of IL-1*β* expression in perturbed chondrocytes may ameliorate degradation of the ECM by helping to restore normal physiologic balance in cartilage, thereby providing a therapeutic option for OA joints. Recombinant adenoassociated virus (rAAV) vectors are ideal candidates for intra-articular gene therapy as they can invade nondividing cells and are nonpathogenic and replication deficient [17]. Transduction of articular tissues, including chondrocytes, synoviocytes, and intact cartilage, by rAAV vectors has been well demonstrated [18–20].

The objective of this study was to evaluate the effects of rAAV2 mediated knockdown of IL-1*β* on gene expression and protein synthesis in chondrocytes cultured in an OA model. We hypothesized that transduction of chondrocytes with rAAV2-shIL-1*β* prior to stimulation with lipopolysaccharide (LPS) would effectively silence IL-1*β* and would in turn decrease expression of catabolic cytokines, degradative enzymes, and inflammatory mediators involved in the IL-1*β* signaling pathway, compared to untransduced chondrocytes. We also hypothesized that IL-1*β* interference would rescue suppression of key matrix proteins, including collagen type II and aggrecan, which occurs following LPS stimulation.

## 2. Materials and Methods

### 2.1. Tissue Culture

Cartilage was harvested from the articular surface of three young horses (<2 years) using a protocol approved by the Institutional Animal Care and Use Committee and digested in 0.075% collagenase (Worthington Biochemical, Lakewood, NJ) as previously described [21]. Following digestion, cells were filtered and centrifuged at 300 ×g for 10 minutes. Cell pellets were washed and cells counted before plating in 24-well plates (Corning Inc., Corning, NY) at a density of 1 × 10^5^ cells/cm^2^. Chondrocytes were cultured in Ham's F12 medium (Gibco, Waltham, MA) supplemented with 10% fetal bovine serum (FBS), 50 *μ*g/mL ascorbic acid, 30 *μ*g/mL *α*-ketoglutarate, 300 *μ*g/mL L-glutamine, 100 units/mL penicillin/streptomycin, and 25 mM HEPES (Gibco, Life Technology, Grand Island, NY) and allowed to adhere for 48 hours. Prior to any transfection or transduction procedure, medium was replaced with serum-free Opti-MEM (Invitrogen, Grand Island, NY) for 4 hours. All chondrocyte monolayer experiments were performed in triplicate.

### 2.2. siRNA and Plasmid Screening

Three siRNA sequences targeting IL-1*β* were designed by Ambion (Grand Island, NY) using their proprietary software and three siRNA sequences were designed using the online algorithm at the Public TRC Portal (http://www.broadinstitute.org/rnai/public/seq/search) (Table 1). These six siRNA sequences were synthesized (IDT, Coralville, IA) and evaluated for knockdown efficiency in monolayer cultures using chondrocytes from 3 different horses. Cells were transfected with siRNA in serum-free medium using DharmaFECT transfection reagent (Thermo Scientific, Waltham, MA). Following transfection, chondrocytes were stimulated with 50 *μ*g/mL* E. Coli* O55:B5 lipopolysaccharide (LPS; Sigma-Aldrich, St. Louis, MO) in serum-free medium. After 24 hours cells were lysed and RNA was isolated using the PerfectPure RNA kit (5 Prime, Gaithersburg, MD). The two siRNA sequences with the most profound decrease in IL-1*β* expression were then designed as short hairpins (sh) by addition of a loop, poly A tail, and restriction sites. The shRNA sequences were synthesized as dsDNA (IDT, Coralville, IA) and ligated into the U6 promoter-driven pSilencer 2.1-U6 Puro plasmid (Life Technologies, Grand Island, NY). Knockdown efficiency of the 2 shRNA sequences was determined in monolayer chondrocytes following transfection of cells using FuGENE HD transfection reagent (Promega, Madison, WI).

### 2.3. Adenoassociated Viral Vector Production

The most efficient shIL-1*β* sequence and the red fluorescent protein, tdTomato (tdT), were subcloned into the rAAV transfer plasmid pHpa-trs-SK. The transgenes were flanked by inverted terminal repeats; tdT was under control of the cytomegalovirus (CMV) early promoter and shIL-1*β* was under control of the U6 promoter. Self-complementary rAAV2-tdT-shIL-1*β* vector was generated by the Research Vector Core at the Children's Hospital of Philadelphia (CHOP) in HEK293 cells using the triple plasmid transfection method, as described [22]. AAV vectors were purified by density gradient centrifugation and viral titers were determined using quantitative dot blot.

### 2.4. AAV Transduction and LPS Stimulation of Chondrocytes

Chondrocytes were cultured in monolayer as described above for 48 hours. Four treatment groups were evaluated: (1) no vector, no LPS (C); (2) no vector, LPS (LPS); (3) rAAV2-tdT-shIL-1*β*, no LPS (shIL-1*β*); and (4) rAAV2-tdT-shIL-1*β*, LPS (shIL-1*β* + LPS). Medium was changed to serum-free Opti-MEM 4 hours prior to transduction. Chondrocytes were transduced with 1 × 10^5^ viral genomes (vg)/cell for 2 hours at 37°C. Transduction medium was then replaced with fresh Opti-MEM and cultures were maintained for 48 hours prior to LPS stimulation. Chondrocytes were stimulated with 50 *μ*g/mL of LPS for 24 hours prior to collection and further analysis.

### 2.5. Flow Cytometry and Fluorescent Microscopy

Chondrocytes were trypsinized, washed, and suspended in 0.5% PBS/BSA 24, 48, and 72 hours after transduction for flow cytometric analysis using a FACSCanto II flow cytometer (BD Biosciences, San Diego, CA). tdTomato expression was quantified using a 585/42 filter to measure fluorescence. Untransduced chondrocytes were used to set fluorescent gates in order to account for autofluorescence. Fluorescent microscopy, to visually assess transduction of cells, was also performed at these time points.

### 2.6. Gene Expression

Chondrocytes were lysed and RNA was isolated using the PerfectPure RNA Kit (5 Prime, Gaithersburg, MD). Purity and concentration of the RNA were assessed using UV microspectrophotometry (NanoDrop 2000 Spectrophotometer, Thermo Scientific, Waltham, MA). Gene expression was quantified by real-time quantitative polymerase chain reaction (RT-qPCR) using the Taqman One-Step RT-PCR technique with all samples run in duplicate (ABI PRISM 7900 HT Sequence Detection System, Applied Biosystems, Foster City, CA). Equine primers and dual-labeled fluorescent probes [6-carboxyfluorescein (FAM) as the 5′ label (reporter dye) and 5-carboxymethyl rhodamine (TAMRA) as the 3′ label (quenching dye)] were designed using Primer Express Software Version 2.0 (Applied Biosystems, Foster City, CA) (Table 2). All primers and probes were designed using equine specific sequences published in GenBank (National Institutes of Health, Bethesda, MD). Total copy number of mRNAs was determined using absolute quantitative PCR derived from a standard curve developed for each gene at the time of analysis, and these values were normalized to the reference gene, 18S. The efficiency of the primers and probe was determined by evaluating the slope of the log-linear phase and *r* ^2^ values of the standard curve.

### 2.7. PGE_2_ Synthesis

Supernatants were collected from monolayer cultures 24 hours following LPS stimulation and PGE_2_ concentration was quantified using a competitive enzyme immunoassay (Enzo Life Sciences, Farmingdale, NY). Optical density was measured using a Tecan microplate reader at 405 nm with wavelength correction set at 570 nm.

### 2.8. Statistics

Flow cytometry data was expressed as mean percentage ± SEM. Changes in gene expression were shown as mean percentage change ± SEM relative to LPS chondrocytes. Histograms were visually inspected for a Gaussian distribution and Shapiro-Wilk test was performed to confirm normal distribution prior to statistical analysis. Statistical analysis was performed using a one-way analysis of variance (ANOVA) to compare differences between the four different treatment groups. Pairwise comparisons were made using Tukey's* post hoc* test. Statistical analysis was performed using JMP. The level of significance was set at *p* < 0.05.

## 3. Results

### 3.1. siRNA Screening and Vector Development

**Figure 1:**
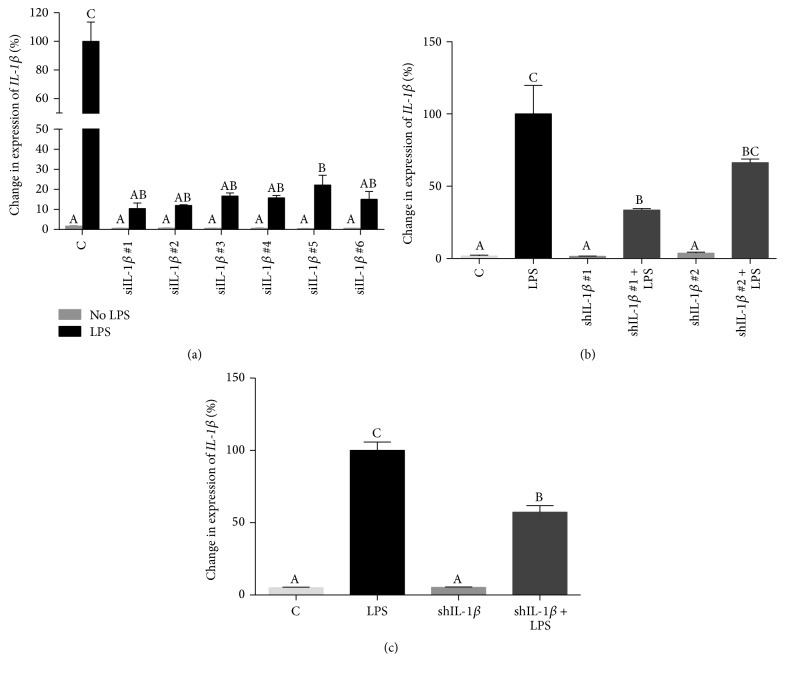
Changes in expression of* IL-1β* in chondrocytes 24 hours following exposure to siRNA and stimulation with LPS (50 *μ*g/mL). % change (±SEM) is shown relative to control chondrocytes treated with LPS. (a) Comparison of six different IL-1*β* targeting sequences, all of which show significant decrease in expression. (b)* IL-1β* expression in LPS-stimulated chondrocytes following transfection with pSilencer expressing shIL-1*β* #1 and shIL-1*β* #2. (c)* IL-1β* expression in LPS-stimulated chondrocytes following transfection with the rAAV transfer plasmid coexpressing tdTomato. Data were analyzed using ANOVA followed by Tukey's* post hoc* pairwise analysis. Graph bars with different Tukey classification letters are significantly different at *p* < 0.05.

Six different sequences targeting IL-1*β* were evaluated for knockdown efficiency in chondrocyte monolayer cultures. All 6 sequences led to significantly decreased expression of* IL-1β* 24 hours following stimulation with LPS (50 *μ*g/mL) (Figure 1(a)); however, sequences #1 and #2 showed slightly superior silencing. Both sequences showed efficient* IL-1β* silencing following ligation into the plasmid pSilencer and transfection of cells using FuGENE HD (Figure 1(b)). Sequence #1 appeared to have superior silencing compared to sequence #2 (Figure 1(b)); therefore, sequence #1 was selected as the shRNA to be expressed by the rAAV vector. Continued efficient knockdown of* IL-1β* was observed following ligation of shIL-1*β* #1 into the AAV transfer plasmid which was designed to coexpress tdTomato (Figure 1(c)).

### 3.2. Transduction Efficiency of rAAV2-tdT-shIL-1*β* in Equine Chondrocytes Cultured in Monolayer

**Figure 2:**
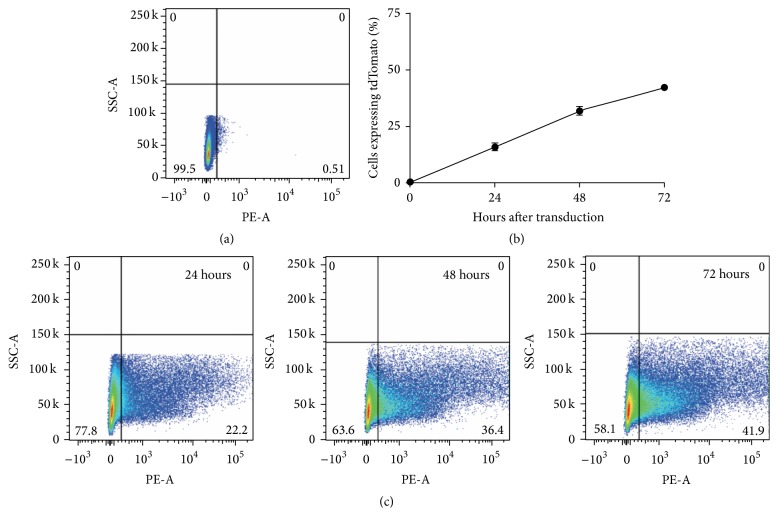
Transduction efficiency of rAAV2-tdT-shIL-1*β* into chondrocytes cultured in monolayer. Fluorescence was quantified using flow cytometry and visually assessed using fluorescence microscopy. (a) Representative flow cytometric plot of untransduced chondrocytes used to set fluorescent gates to account for autofluorescence of chondrocytes. (b) Transduction efficiency assessed by % of chondrocytes expressing the fluorescent protein tdTomato 24, 48, and 72 hours after transduction. (c) Representative flow cytometric plots of transduced chondrocytes 24, 48, and 72 hours following transduction.

**Figure 3:**
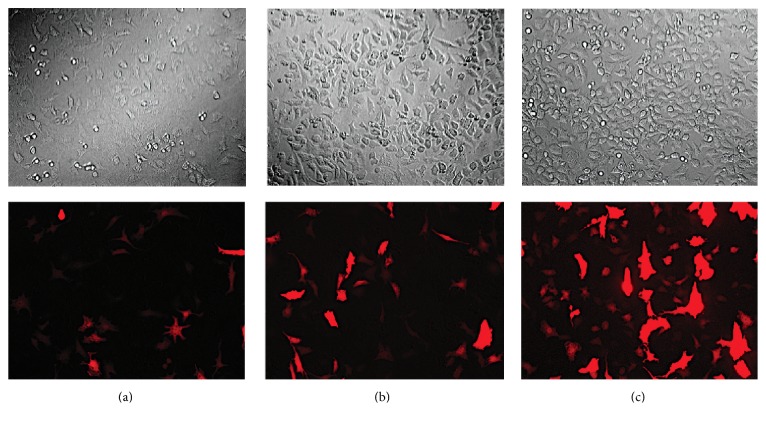
Representative bright field (top) and fluorescence microscopy (bottom) images of chondrocytes cultured in monolayer (a) 24 hours, (b) 48 hours, and (c) 72 hours after transduction with rAAV2-tdT-shIL-1*β*. Fluorescence intensity and number of cells expressing the fluorescent protein increased during the 72-hour culture period. Magnification, 200x.

The red fluorescent protein, tdTomato, was packaged into the rAAV2 vector in order to assess transduction efficiency. Equine chondrocytes cultured in monolayer were efficiently transduced with the rAAV2-tdT-shIL-1*β* vector, using flow cytometry to quantify expression of tdTomato (Figures 2(a)–2(c)). The percentage of cells (±SEM) expressing tdTomato increased throughout the 72-hour culture period following transduction. At 24 hours after transduction, 16 ± 1.66% of cells were positive; at 48 hours after transduction, 31.9 ± 2.0% of cells were positive; and at 72 hours after transduction, 42.2 ± 1.44% of cells were positive for the reporter protein (Figures 2(b) and 2(c)). Fluorescence microscopy images taken at similar time points supported the flow cytometry findings (Figure 3). The number of cells expressing tdTomato increased over the 72-hour culture period.

### 3.3. Effect of rAAV2-tdT-shIL-1*β* Transduction on Expression of Catabolic Cytokines and Degradative Enzymes

**Figure 4:**
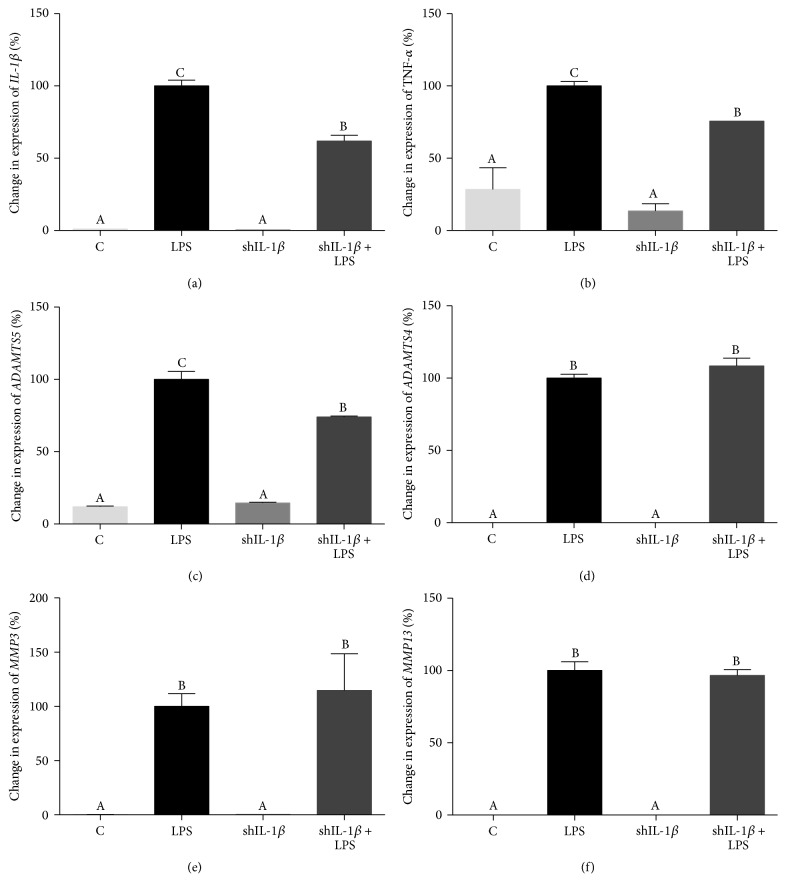
Quantitative polymerase chain reaction (qPCR) data showing % change in gene expression of catabolic cytokines and degradative enzymes in control, untransduced (C), LPS-stimulated (LPS), rAAV2-tdT-shIL-1*β* transduced (shIL-1*β*), and rAAV2-tdT-shIL-1*β* transduced and LPS-stimulated chondrocytes (shIL-1*β* + LPS). % change (±SEM) is shown relative to LPS chondrocytes. shIL-1*β* + LPS chondrocytes had significantly decreased* IL-1β* (a),* TNF-α* (b), and* ADAMTS5* expression (c) compared to LPS chondrocytes. Expression of (d)* ADAMTS4*, (e)* MMP3*, and (f)* MMP13* showed no significant differences between shIL-1*β* + LPS and LPS chondrocytes. Data were analyzed using ANOVA followed by Tukey's* post hoc* pairwise analysis. Graph bars with different Tukey classification letters are significantly different at *p* < 0.05.

Expression of catabolic cytokines and degradative enzymes, including* IL-1β*, tumor necrosis factor-*α* (*TNF-α*),* MMP3*,* MMP13*,* ADAMTS4*, and* ADAMTS5*, was evaluated in rAAV2-tdT-shIL-1*β* transduced and untransduced chondrocyte cultures 24 hours following LPS stimulation. Changes in expression of all genes were evaluated relative to LPS-stimulated chondrocytes (LPS). All catabolic cytokines and enzymes were significantly increased in LPS cultures compared to cultures without LPS stimulation (Figure 4).* IL-1β* expression was significantly decreased in shIL-1*β* + LPS chondrocytes compared to LPS conditioned naïve chondrocytes, 24 hours following LPS stimulation (*p* < 0.0001) (Figure 4(a)), indicating effective IL-1*β* RNA knockdown.* IL-1β* expression was reduced by approximately 42% in shIL-1*β* + LPS chondrocytes but remained significantly higher than control and shIL-1*β* transduced chondrocytes cultured without LPS.* TNF-α* expression was also significantly reduced in shIL-1*β* + LPS chondrocytes compared to LPS conditioned naïve chondrocytes, 24 hours following LPS stimulation (*p* = 0.017) (Figure 4(b)), as was expression of* ADAMTS5* (*p* = 0.0001) (Figure 4(c)). There were no significant differences in expression of* ADAMTS4*,* MMP3*, or* MMP13* in shIL-1*β* + LPS chondrocytes and LPS exposed naïve chondrocytes (Figures 4(d)–4(f)).

### 3.4. Effect of rAAV2-tdT-shIL-1*β* Transduction on Expression of Proinflammatory Mediators

**Figure 5:**
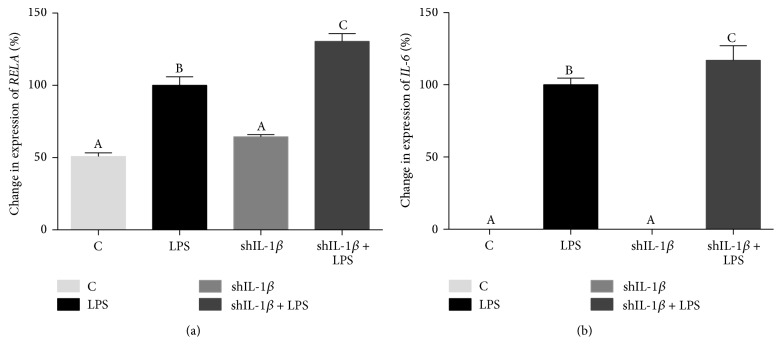
Quantitative polymerase chain reaction (qPCR) data showing % change in gene expression of inflammatory mediators in control, untransduced (C), LPS-stimulated (LPS), rAAV2-tdT-shIL-1*β* transduced (shIL-1*β*), and rAAV2-tdT-shIL-1*β* transduced and LPS-stimulated chondrocytes (shIL-1*β* + LPS). % change (±SEM) is shown relative to LPS chondrocytes. Expression of (a)* RELA* (p65) and (b)* IL-6* was significantly increased with LPS stimulation. The expression of both (a)* RELA* (p65) and (b)* IL-6* was highest in shIL-1*β* + LPS chondrocytes. Data were analyzed using ANOVA followed by Tukey's* post hoc* pairwise analysis. Graph bars with different Tukey classification letters are significantly different at *p* < 0.05.

In addition to the major catabolic cytokines and degradative enzymes in articular cartilage, changes in expression of several inflammatory mediators were evaluated. Expression of* RELA* (p65), a major subunit in the NF-*κ*B family of transcription factors, was significantly increased by LPS stimulation in both LPS and shIL-1*β* transduced LPS conditioned chondrocytes compared to unstimulated chondrocytes (Figure 5(a)). Additionally,* RELA* (p65) expression in shIL-1*β* + LPS chondrocytes was even higher than that in LPS chondrocytes (*p* = 0.005).* IL-6* expression was significantly increased by LPS stimulation in both untransduced and transduced chondrocytes (Figure 5(b)). Similar to the pattern seen with* RELA* (p65),* IL-6* expression in shIL-1*β* + LPS chondrocytes was significantly higher than that in LPS chondrocytes (*p* = 0.01).

**Figure 6:**
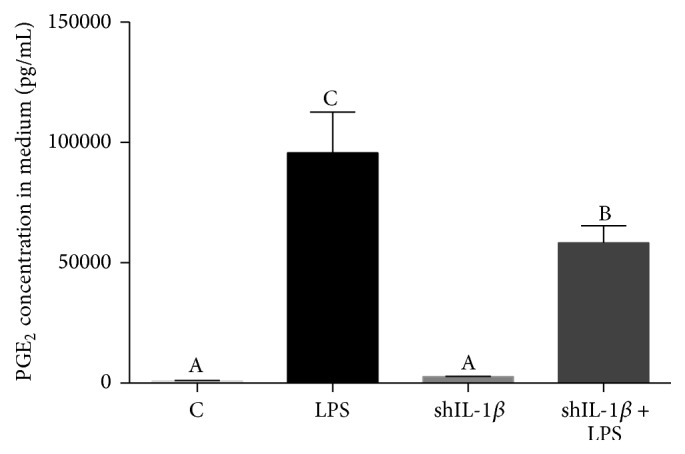
PGE_2_ concentration in medium collected from chondrocyte cultures 24 hours after stimulation with LPS. Chondrocytes transduced with rAAV2-tdT-shIL-1*β* had significantly decreased PGE_2_ in the medium compared to LPS chondrocytes. Data were analyzed using ANOVA followed by Tukey's* post hoc* pairwise analysis. Graph bars with different Tukey classification letters are significantly different at *p* < 0.05.

Supernatants from chondrocyte cultures were collected 24 hours following LPS stimulation and PGE_2_ concentrations were quantified using ELISA. PGE_2_ was significantly increased in LPS-stimulated chondrocytes compared to unstimulated chondrocytes. However, PGE_2_ was significantly decreased in shIL-1*β* transduced chondrocytes exposed to LPS compared to LPS conditioned naïve chondrocytes (*p* = 0.029) (Figure 6).

### 3.5. Effect of rAAV2-tdT-shIL-1*β* Transduction on Expression of ECM Proteins

**Figure 7:**
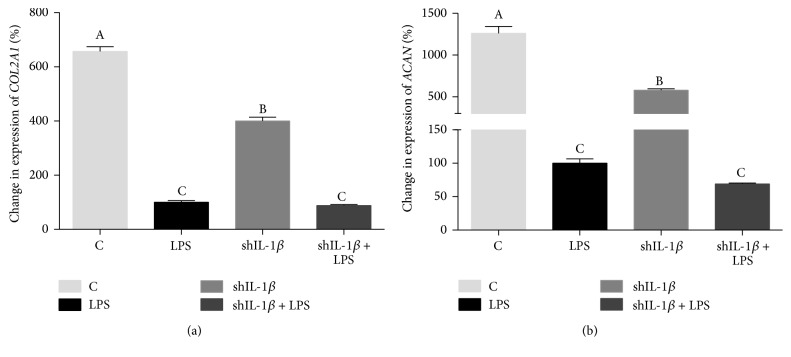
Quantitative polymerase chain reaction (qPCR) data showing % change in gene expression of ECM proteins in control, untransduced (C), LPS-stimulated (LPS), rAAV2-tdT-shIL-1*β* transduced (shIL-1*β*), and rAAV2-tdT-shIL-1*β* transduced and LPS-stimulated chondrocytes (shIL-1*β* + LPS). % change (±SEM) is shown relative to LPS chondrocytes. (a)* COL2A1* and (b)* ACAN* are shown. Both* COL2A1* expression and* ACAN* expression are decreased with LPS stimulation and this is not rescued with rAAV2-tdT-shIL-1*β* transduction. Data were analyzed using ANOVA followed by Tukey's* post hoc* pairwise analysis. Graph bars with different Tukey classification letters are significantly different at *p* < 0.05.

Expression of the major ECM proteins, aggrecan (*ACAN*) and collagen type II (*COL2A1*), was also evaluated in rAAV2-tdT-shIL-1*β* transduced and untransduced chondrocyte cultures 24 hours following LPS stimulation. Changes in expression of all genes were evaluated relative to LPS conditioned chondrocytes. LPS stimulation caused significant suppression of both* COL2A1* expression and* ACAN* expression (Figures 7(a) and 7(b)). Expression of* COL2A1* was highest in control (C) chondrocytes (Figure 7(a)). Interestingly,* COL2A1* expression was suppressed in shIL-1*β* chondrocytes that were transduced only (no LPS stimulation) compared to control chondrocytes (*p* < 0.0001) (Figure 7(a)). Additionally, the suppression of* COL2A1* in LPS chondrocytes was not rescued by IL-1*β* silencing. A similar pattern was noted in expression of* ACAN*; shIL-1*β* chondrocytes had lower* ACAN* expression than control chondrocytes (*p* < 0.0001) (Figure 7(b)). Again,* ACAN* suppression seen in LPS chondrocytes was not rescued by IL-1*β* silencing.

### 3.6. PCR Efficiency

The slope of the log-linear phase and *r* ^2^ values showed efficient amplification of the primers for all genes evaluated. Slopes ranged from −3.0 to −3.4 and *r* ^2^ = 0.99. The efficiency of the PCR was calculated to be between 96% and 115% (efficiency = −1 + 10^(−1/slope)^).

## 4. Discussion

Posttranscriptional silencing of IL-1*β* with an rAAV2 vector expressing a short hairpin sequence targeting equine IL-1*β* led to effective reduction in IL-1*β* in chondrocytes stimulated with LPS. To the authors' knowledge, this is the first report of knockdown of a pivotal catabolic cytokine in the pathogenesis of OA, in equine chondrocytes, using an rAAV vector expressing a shRNA. IL-1*β* interference also had significant downstream effects on a major degradative enzyme,* ADAMTS5*, and catabolic cytokine,* TNF-α*. Additionally, supernatants from LPS-stimulated chondrocytes transduced with rAAV2-tdT-shIL-1*β* had significantly less PGE_2_ than untransduced, LPS-stimulated chondrocytes. Despite alterations in several factors involved in degradation of the ECM and pathogenesis of OA, IL-1*β* silencing in monolayer cultures did not rescue suppression of ECM components, including collagen type II and aggrecan, observed following LPS stimulation.

The rAAV2-tdT-shIL-1*β* vector was able to effectively transduce chondrocyte cultures. As expected, transduction efficiency increased over the 72-hour culture period, although at 72 hours following transduction, only 42% of cells were positive for the reporter protein, tdTomato. Similar studies evaluating rAAV2 mediated transduction of chondrocytes have shown higher efficiencies (~96%) 7 days following transduction [23]; therefore, it is likely that the percentage of cells expressing tdTomato would have continued to increase past the 72-hour culture period used in this study. Cells were stimulated with LPS 48 hours after transduction when approximately 30% of cells were positive for expression of tdTomato which may explain the moderate knockdown (~40%) of IL-1*β* observed. Chondrocytes have been shown to quickly dedifferentiate when cultured in monolayer [24]; therefore, it can be difficult to establish an ideal culture length where chondrocytes maintain their phenotype but are able to reach peak transduction. The use of three-dimensional chondrocyte culture systems in which chondrocytes maintain their phenotype would allow for longer term evaluation of transduction and the effects of IL-1*β* knockdown. The gene expression data obtained from monolayer cultures in this study provide proof of principle and support the extension of this IL-1*β* knockdown into 3D culture systems.

By targeting IL-1*β*, a key cytokine in the catabolic cascade, expression of the degradative enzyme (*ADAMTS5*) and catabolic cytokine (*TNF-α*) activated by IL-1*β* were simultaneously decreased. Although both MMPs and aggrecanases are involved in breakdown of the ECM, aggrecanases likely play a more dominant role [25]. Within the aggrecanases family, ADAMTS5 has been shown to be the major aggrecanase in murine and equine cartilage degeneration [26, 27]. Decreased expression of* TNF-α* in rAAV2-tdT-shIL-1*β* transduced chondrocytes is also encouraging because, along with IL-1*β*, TNF-*α* plays a central role in joint inflammation and degradation [28]. Unfortunately, similar decreases in expression of other downstream factors including* MMP3*,* MMP13*, and* ADAMTS4* were not found with IL-1*β* knockdown. The differences in downstream effects following IL-1*β* knockdown likely reflect the complex regulatory pathways at play.

Although rAAV2-tdT-shIL-1*β* led to suppression of several important mediators of OA, the vector was also associated with a proinflammatory response. NF-*κ*B is a dimeric transcription complex made of several different proteins and is a central regulator of cellular inflammation [29]. RELA (p65) is a major subunit involved in NF-*κ*B activation which causes upregulation in expression of several proinflammatory cytokines including IFN-*γ*, TNF-*α*, and IL-6 [30]. In this study, LPS stimulation led to significant upregulation of* RELA* (p65) expression. Interestingly,* RELA* (p65) expression was even higher in shIL-1*β* + LPS chondrocytes compared to untransduced chondrocytes exposed to LPS. A similar pattern was observed for expression of* IL-6*. Such alterations in these proinflammatory genes were not observed in transduced chondrocytes that were not stimulated with LPS which suggests an interactive effect of rAAV2-tdT-shIL-1*β* transduction and LPS stimulation. Possible explanations for increased expression of* RELA* (p65) and* IL-6* include an intracellular immune response to AAV or to the shRNA which is produced. Both AAV and shRNA duplexes are capable of activating an intracellular immune response through TLR-9 or TLR-7 signaling, respectively [31, 32]. Additionally, off-target silencing and undesirable alterations in gene expression have been shown to be side effects of shRNA treatment [33]. Evaluation of an AAV vector with a nontargeting shRNA sequence and an AAV vector without a shRNA sequence would have provided important information regarding the cellular immune response. Temporal gene expression patterns as well as a more complete evaluation of the transcriptome would also shed light on the effects of rAAV2-tdT-shIL-1*β* transduction in equine chondrocytes. It is important to note that although increases in* RELA* (p65) expression suggest activation of the NF-*κ*B pathway, further evidence including phosphorylation of RELA (p65) and nuclear translocation of the complex are needed to confirm this. Further investigations into the immune response to intracellular AAV and shRNA will aim to address this.

Knockdown of IL-1*β* in stimulated chondrocytes failed to rescue the suppressed matrix protein expression caused by LPS. Expression of aggrecan and collagen type II, the major components of the ECM, was significantly decreased following LPS stimulation and this effect was not altered by rAAV2-tdT-shIL-1*β* transduction. In fact, transduction alone appeared to decrease production of ECM proteins. It is possible that an intracellular immune response to the rAAV vector and/or the shRNA sequence led to these changes; however, further experiments are required to elucidate these potential responses. Monolayer culture of chondrocytes results in a steady decline in the chondrocyte phenotype with loss of ECM production and this was exacerbated by transduction, LPS, and both transduction and LPS exposure. These effects suggest that the monolayer model is an effective screening tool only for short-term cultures and that 3D suspension or explant cultures will be required to verify ECM matrix rebound after RNA silencing of catabolic cytokines.

A limitation in this study is the lack of protein quantification by ELISA or Western blot to corroborate changes in gene expression. Unfortunately, no commercial antibodies cross-react with equine IL-1*β*, TNF-*α*, MMPs, or the aggrecanases, and equine-specific antibodies to these proteins have not been successfully developed (B. Wagner, unpublished data). An exhaustive evaluation of commercially available anti-IL-1*β* antibodies was performed without success. Additionally, several unsuccessful attempts to develop an equine-specific anti-IL-1*β* antibody have been made (B. Wagner, unpublished data). Equine IL-1*β* is a seemingly minimally antigenic protein in mice, which limits the ability to develop an equine-specific monoclonal antibody. The downstream effects noted in TNF-*α*, ADAMTS5, and PGE_2_ suggest that posttranscriptional silencing of IL-1*β* leads to decreased production of the functional IL-1*β* protein.

Overall, rAAV-mediated posttranscriptional silencing of IL-1*β* provided some protection of chondrocytes following perturbation with LPS, a common model of OA,* in vitro*. Transduction of chondrocytes in monolayer with the rAAV2-tdT-shIL-1*β* vector led to decreased expression of* IL-1β*, which in turn downregulated expression of* ADAMTS5* and* TNF-α*, and decreased production of PGE_2_. These results suggest that RNAi targeting IL-1*β* may be a feasible method for manipulating the transcriptome in chondrocytes following injury, thereby altering the catabolic cascade that follows and limiting cartilage degradation and development of OA. A gene therapy approach using an AAV vector would allow for effective and sustainable intra-articular expression of a targeting shRNA. It is important to note that, in this monolayer study, knockdown of IL-1*β* did not lead to suppression of all degradative enzymes and catabolic cytokines and failed to rescue suppression of expression of ECM proteins; therefore, a global protective effect may not occur* in vivo*. In addition, despite the encouraging alterations in chondrocyte gene expression following IL-1*β* knockdown, the apparent increase in the expression of inflammatory mediators* RELA* (p65) and* IL-6* in rAAV2-tdT-shIL-1*β* transduced chondrocytes warrants further investigation before AAV-mediated RNAi could be considered for clinical use.

## Figures and Tables

**Table 1:** siRNA sequences targeting IL-1*β*.

siIL-1*β* sequence name	Sequence
#1	5′-CCAGUGACAUGAUGACUUA-3′
#2	5′-UCCGGGACAUAUACCAUAAAU-3′
#3	5′-GCUUCAAUUCUCCCACCAA-3′
#4	5′-GACAACUGGGAUGAUGAUUAU-3′
#5	5′-AAGUCAGUUAUGUCCCGGCCG-3′
#6	5′-CCAGUUUAAUUUGGACUAG-3′

**Table 2:** Equine primer sequences used to analyze gene expression^*∗*^.

18S	Forward, 5′-CGGCTTTGGTGACTCTAGATAACC-3′
	Reverse, 5′-CCATGGTAGGCACAGCGACTA-3′

IL-1*β*	Forward, 5′-CGTCTCCCAGAGCCAATCC-3′
	Reverse, 5′-CACCAGGCTGACTTTGAGTGAGT-3′

TNF-*α*	Forward, 5′-CAGCCGCTTAGCTGTCTCCTA-3′
	Reverse, 5′-GTGTGGCAAGGGCTCTTGAT-3′

ADAMTS4	Forward, 5′-CCCTGGTCTCCGAAACCTCTA-3′
	Reverse, 5′-TATTCACCATTGAGGGCATAGGA-3′

ADAMTS5	Forward, 5′-CAGACGTTGGGACCATATGCT-3′
	Reverse, 5′-TGCGTGGAGGCCATCAT-3′

MMP3	Forward, 5′-CTTATCAAAAATGGCTGCGTCTATT-3′
	Reverse, 5′-GCAGAGACAGTGTTTTCATTTTTAAG-3′

MMP13	Forward, 5′-TGAAGACCCGAACCCTAAACAT-3′
	Reverse, 5′-GAAGACTGGTGATGGCATCAAG-3′

IL-6	Forward, 5′-AGTAACCACCCCTGACCCAACT-3′
	Reverse, 5′-TGTTGTGTTCTTCAGCCACTCA-3′

RELA (p65)	Forward, 5′-GCTTATGGATTCTGAGGGTGTGT-3′
	Reverse, 5′-CCAAAAGGATATAGATACTGCCAATAAA-3′

Collagen type II	Forward, 5′-CGCTGTCCTTCGGTGTCA-3′
	Reverse, 5′-CTTGATGTCTCCAGGTTCTCCTT-3′

Aggrecan	Forward, 5′-GATGCCACTGCCACAAAACA-3′
	Reverse, 5′-GATGCCACTGCCACAAAACA-3′

^*∗*^18S, 18 small ribonucleic acid; IL, interleukin; TNF-*α*, tumor necrosis factor-*α*; ADAMTS, a disintegrin and metalloproteinase with thrombospondin motifs; MMP, matrix metalloproteinase.
